# The Cause of the Failure

**Published:** 1905-12

**Authors:** 


*President.*

The foregoing important notice from the President of the International Dental Publication Company is self-explanatory and requires no further comment. This journal closes its eighteen years of independent life with this number.

### THE CAUSE OF THE FAILURE.

Many of our readers connected with the National Dental Association will be mystified upon reading the announcement of the suspension of the International Dental Journal, for they probably left the meeting at Buffalo, August last, with the full expectation that this journal would be transferred to the national organization and be continued, with some modification as to name, as its organ. They had reason to expect this, for the International had been offered free of cost by the International Dental Publication Company, provided the National Association would continue its publication in entire independence of all commercial entanglements. By a unanimous vote this proposition was accepted.

The International Dental Publishing Company had two years previously made this offer and it was held over to give the National Dental Association ample time for its full consideration. Two Presidents of that body, Dr. Noel, at Asheville, and Dr. Boardman, at Buffalo, had both advocated the transfer and the publication of a journal representing the National Association. The former appointed, at the Asheville meeting, a committee to take the matter in charge, of which the writer was chairman.

This committee made a favorable report at the Buffalo meeting and the subject was referred to the Executive Council. This body appointed a special evening for the consideration of the subject, at which prominent members of the national organization were invited to be present. At that meeting the entire subject of establishing a journal was very freely discussed. The only dissenting voices heard were not based in opposition to the proposition as presented, but the fear was expressed that its adoption would involve the Association in financial difficulties. This objection was met by the writer with the fact, that while there would be financial responsibility involved in consummating the transaction, the INTERNATIONAL DENTAL JOURNAL had for eighteen years met its own business expenses, with no year showing a deficit, and that therefore the financial risk to the Association was limited to a slight increase in expense, due to the transfer and to more extended editorial supervision. To meet this possible increase, contributions of quite large amounts were offered voluntarily by several members. After a lengthy consideration of the subject the Council felt itself unequal to deciding the question and by vote directed it be sent back to the general body with a favorable recommendation.

This was on Thursday evening, July 27th. That same evening the Secretary, Dr. Peck, states that the President of Council sent to the meeting of the general body that the "Executive Council recommends that the proceedings for the year be given to the *Dental Cosmos* for publication, which, on motion, was adopted." This fact was unknown to the writer at the time and presumably no one of the others interested, outside of the Council, was aware that such action had been taken. It will be remembered that the two bodies, the Executive Council and general meeting, were in session at the same time and the same evening. Those invited to be present were not informed that action had been taken in regard to the proceedings. This was all kept *sub rosa* and, as far as the writer was concerned, he was not aware of this action for weeks subsequently.

In ignorance of the aforesaid action, the writer, with others, went to the general meeting on Friday morning and then and there the report of the Executive Council favoring the establishing of a dental journal and the acceptance of the offer of the INTERNATIONAL DENTAL JOURNAL was received, and without discussion was unanimously adopted. The President subsequently appointed a committee of five to carry out the proposed measure, with full powers to act.

This appointment carried with it not only power to arrange the necessary procedures in starting the journal, but to secure the necessary funds to meet a possible deficit the first year. Its appointment took from the Executive Council, or the National Dental Association, the power to sell or otherwise dispose of the proceedings for the year 1905. These belonged by right of appointment to the committee. When this was named by the President, the Executive Council should have reconsidered its action in regard to the proceedings and handed these to the proper custodian, the chairman of the Committee on Journal. This was not done and the reason given why this was not even suggested was that "nothing definite would be done by that committee until the next meeting of the National Dental Association." Why the Council should have decided what the Committee on Journal could or could not do will remain an unsolved problem as far as the writer is concerned.

Whether the Committee on Journal met for organization at Buffalo is unknown. It is presumed it did not, possibly having heard of the action in regard to the proceedings. It is certainly true that nothing was heard from this committee until quite late in October. It had up to that time accomplished nothing. From what the writer has been able to learn the committee had arrived at the conclusion that the action in limiting membership in the national body to State societies would cause such a marked diminution of membership that a journal could not find proper support, and further, that the giving the proceedings to another journal made any action impossible for the present year.

That the committee has laid itself open to criticism is beyond question. It should have insisted on its rights in the premises and organized at once for active service. The managers of the INTERNATIONAL DENTAL JOURNAL were prepared to meet the members of said committee, but no official communication was received at any time as to the action proposed to be taken. The management of the INTERNATIONAL was obliged to practically force the committee to come to some decision and this, as before stated, came very late in October.

This action, or rather lack of action, made it necessary for the International Dental Publication Company to act at once, and at a meeting of the Board of Directors the offer previously made to transfer the INTERNATIONAL DENTAL JOURNAL to the National Dental Association was withdrawn.

The writer does not care to comment upon the peculiar action of all concerned in the national body. He regards the mistakes, if such they were, as very serious. It seems to involve the very life of the National Association, and unless active measures be taken to bring that organization into line with true professional progress its decline and death are assured. The dental profession cannot afford to stand still; it must advance with the spirit of the age, and if present organizations are unfaithful they must give place to others more truly representative of the professional spirit.

